# Knowing What You Need But Not What You Want: Affordances and Action-Defined Templates in Neglect

**DOI:** 10.1155/2002/635685

**Published:** 2002-10-01

**Authors:** Glyn W. Humphreys, M. Jane Riddoch

**Affiliations:** Behavioural Brain Sciences Centre School of Psychology University of Birmingham Birmingham B15 2TT UK

## Abstract

We examined search for target objects in a patient, MP, showing symptoms of left unilateral neglect. The conditions varied how the target was defined, the numbers of targets and distractors, and whether search was for multiple or single targets. We found that search was substantially improved when the target was defined by a description of its action rather than its name. This advantage for action-defined targets increased with larger display sizes. For both action and name-defined targets, there were also larger effects of the number of distractors when search was for multiple rather than single targets, even when the numbers of distractors were kept constant. However, these effects were increased for name-defined targets. The differences between action- and name-defined targets decreased when objects were replaced with words. The data suggest that search could be based on action-defined templates of targets, activated by affordances from objects. The action-defined templates facilitated detection and reduced MP's tendency to re-search displays.

## 1. Introduction

Experimental studies of visual search have typically examined two situations: (i) where a target is defined by the differences between its perceptual properties and those of distractors ('find the odd one out'), and (ii) where a target is pre-specified by its perceptual properties ('find the red T') (see [39], for one review). In the first case, search may operate in a relatively bottom-up manner, determined by salient feature differences between items in the field (though see [1]). In the latter case, search may be modulated by a 'memory template', that biases processing in favour of the target relative to distractors (e.g. [6,9]). For example, this memory template could be 'set up' within the frontal lobes based on the task instructions, with activation then being fed to influence processing within more posterior brain regions (e.g. [24]). In the present study we ask whether search can be influenced by 'memory templates' that are defined not in terms of the particular perceptual properties of a target, but rather the action that the target might perform. An example of this in everyday life might be searching for a container to catch water dripping from a ceiling, where a saucepan, a bowl, or even a cup might suffice. Here you may look for an object that fulfills the functional need of the intended action, without having a complete specification for a particular target. Do such action-defined templates exist, and if so, what forms of information are they activated by? We address these questions by an analysis of a patient, MP, who showed aspects of visual neglect when asked to detect targets in cluttered environments. We demonstrate a dissociation between MP's ability to use action- and name-defined templates in search. When action-defined templates were used, there was both better detection of targets and reduced re-searching of objects. We discuss the role of action-defined templates in search and how such templates may modulate visual neglect.

## 2. Memory templates in search

Studies of visual search in normal human observers demonstrate that performance can be affected by the memory representation held of targets. Neisser [25]

ISSN 0953-4180/01/02/$8.00 © 2001/2002 – IOS Press. All rights reserved noted data showing that search was slowed as the number of potential targets increased, indicating either that memory templates are matched serially against a display or some form of competition between templates when they are matched in parallel. Search reduced in efficiency when a template could not be used optimally to facilitate detection of the target. More direct evidence for a modulatory role of memory templates in search was found by Pashler [26]. He reported an effect of similarity between distractors and items held in memory but not present in the display. Participants had to search for one of two targets (C or E), and distractors could be similar to one of the targets (G or F) or to neither (X or N). Reaction times (RTs) to detect a target amongst dissimilar distractors were slowed if the distractors were nevertheless similar to the other target (held in memory but not in the display), relative to when they were dissimilar to both targets. Here partial matches from distractors to a memory template slowed search.

An even more dramatic result comes from studies of category effects on search. Jonides and Gleitman [21] found that search for an ambiguous O shape amongst distractor letters could be efficient or inefficient depending on whether subjects were asked to detect a number or the letter O; likewise search for the same target amongst distractor numbers was efficient when the instruction was to search for a letter and inefficient when the task was to search for a number O. Similar changes in the efficiency of searching the same displays, according to how targets are categorized, have been noted more recently by Humphreys and Müller [19], using stimuli with reversable figure-ground relations. Search was efficient or inefficient depending on which parts subjects were instructed to encode as 'figure' and which as 'ground'. In these last instances, the instruction as to how to code the template for the target appears to have a large effect on search. In the case of category effects on search, coding a template at a general, categorical level ('find the number amongst letters'), can even lead to search that is more efficient than when the template is coded for the perceptual features of the target ('find the letter O amongst other letters').

A physiological account of how memory templates may influence search was provided by Chelazzi et al. [6]. They cued monkeys with the features of an upcoming target (e.g., a red circle) and trained the animals to make an eye movement to this target when it was presented a short time later with a distractor stimulus. They recorded from cells in the inferotemporal cortex responsive to the features of the target and found that these cells, when cued, remained active during the interval between the cue and the subsequent display. When cued in this way the activation of the 'target' cells was subsequently boosted when the target and distractor were presented together. As a consequence, activity in cells responsive to target features were dominant over cells responsive to the features of distractors, enabling the target to be selected for the eye movement response. Chelazzi et al. suggested that the pre-cued activity of cells responsive to target features provides a memory template for the target, which biases competition between cells activated by targets and distractors. Haenny, Maunsell and Schiller [13] reported similar modulatory effects in area V4, where some cells responded according to the stimulus being sought even when it is not present.

This idea, of search modulated by memory templates for targets, has also been taken up in both mathematical (e.g. [5]) and computational models [16,18,38]. In such models, top-down activation from memory templates can even over-ride bottom-up biases from more salient distractors and from effects of brain lesions that favour some spatial regions over others [16]. Such top-down effects from memory templates can help explain why, for example, patients with visual neglect can be less impaired when processing words than nonwords, even when guessing is controlled [3,20,22,34]. For example, a memory template for a word can help 'fill-in' for degraded input on the contralesional side.

## 3. Activation of actions from affordances: Prior evidence

In the majority of experimental studies, memory templates for search targets have been defined perceptually. However, as we have indicated above, in some real-life examples we may not have a clearly specified perceptual representation of a target, but rather the target may be specified in functional terms ('find an object to catch water from the ceiling'). Can functionally-defined templates influence search? And, if they can, what kind of information might be involved in activating such templates?

We [20] have recently presented evidence relevant to this issue. We reported data from a patient, MP, who showed aspects of unilateral visual neglect, often failing to detect targets presented on the left side of cluttered displays. This problem was apparent when targets were defined by their name, by another functionally equivalent object (e.g., for a cup target a different type of cup was shown as the cue), and by a salient perceptual property ('find the red object'). On these trials, the template for the target was ineffective in helping MP to overcome a spatial deficit in target detection. MP was slow to find targets and he made many omission errors, particularly to targets on the left of the display (though there were also some omissions of targets on the far right). In contrast, performance improved when the target was defined by an action that could be performed with it – using either a verbal definition of an action ('find the object you could drink with') or a gesture (the experimenter gestured a drinking action before the patient started the search task). MP was then faster and more successful at detecting target objects. To account for this result, we proposed that MP's lesion prevented him from using a perceptual template in search. Nevertheless, visual information on the affected side still appeared to be processed and matched to a template defined in terms of the intended action. This is consistent with action-defined templates existing independently of perceptual templates for targets.

One other interesting finding was that the advantage for action-defined templates was eliminated under two circumstances: (i) when words were searched rather than objects, and (ii) when objects were present but had their handles facing away from MP. MP was able both to read the words and to name individual objects correctly, and he showed no deficits in accessing semantic information from these stimuli. Hence the advantage for action-defined templates with object search was unlikely to reflect differences in accessing semantics from objects and words. Instead of this, the advantage may have arisen because objects have privileged access to action templates, when compared with words, due to the visual properties of objects being correlated with their function. J.J. Gibson [12] argued that actions could be based on affordances from objects, contingent on the overlap between the perceptual properties of the objects and the goal of the actor (see the General Discussion for further consideration of the concept of affordances). We suggested that action-defined templates, held by MP, could be activated by affordances from objects presented in the appropriate orientation for the action. The advantage from affordances was thus lost when the orientation of the objects was incongruent with the action and when words replaced objects in the search task. It may be that affordances are transmitted by a pragmatic visual route, independent of the visual processing route leading to recognition and semantic access for known objects (cf. [15]). MP's lesion appeared to disrupt his use of search templates operating through the recognition route (based on the perceptual properties of targets), producing poor search for perceptually-defined targets; nevertheless he remained able to use action-defined templates activated by affordances.

In the present paper, we sought to explore MP's capacity to 'detect by action' in more detail, in order to assess the means by which the action-advantage occurred. Previously we found that defining the target by action both increased the likelihood that targets were detected and the speed of detection [20]. However this facilitation could come about in several ways. One possibility is that action-defined templates change the nature of visual search for this patient (e.g., making him switch from a serial search to a parallel search process); another possibility is that action templates alter the ease with which items were evaluated but the basic search process may remain the same. For example, all objects may be searched serially irrespective of whether action- or name-based templates are used, but the detection threshold for template-defined templates may be lower, facilitating performance. We assessed these possibilities in two experiments in which we manipulated the number of objects in the displays.

In Experiment 1, we varied the number of distractors, using either object or word displays (Experiments 1a and 1b respectively). Effects of the number of distractors on search are a standard measure of search efficiency (e.g. [9,35]). If action-defined templates affect detection but not search then the effects of the templates may be additive with those of the number of distractors. On this account, action-defined templates lower the time taken for (and increase the likelihood of) target detection, but any increases in search time when there are more distractors should be equal for action- and name-defined templates. In contrast, the finding of an interaction between search instruction and number of distractors would be consistent with the templates affecting the search process (e.g., search being more efficient with action-defined templates). We report such an interaction, but only with arrays containing objects. With words there was no advantage for action- over name-defined targets (see also [20]).

Unfortunately, interpretation of such an interaction is not unambiguous. For example, an interaction between search instruction and the number of distractors could also be consistent with a detection-only effect if there is serial search of the objects. If the time taken to match each object against the template is reduced for action-defined templates, then search times will increase less for action-defined search when more objects are in the displays even if the search process itself is constant. For example, the time for a serial search to be conducted on a given display will be based on two factors: the search rate (e.g., the time to select the next item for inspection) and the time to match each item to the template (the 'inspection time'). Since each item inspected increases the total inspection time, so the contribution of this component to search will increase when more items are present. If the inspection time varies according to whether an action or name template is given, then, likewise, the effect of the memory template will increase for larger displays.

To provide a further test of why MP performed differently with name- and action-based instructions, we conducted Experiment 2 in which we varied the number of identical targets whilst keeping constant the number of objects present. Across different trial blocks MP had to search for 1, 2, 3 or 4 targets, in arrays of 10 objects. On a serial search account, search should asymptote as the number of targets increases, since, on average, there will be smaller increases in the number of objects to search as more targets are detected.[1] In contrast to this, we found not only that the difference between name- and action-defined search for objects increased when more targets had to be detected, but there were also exponential rather than asymptotic effects of the number of targets on search. These exponential effects suggest that action-defined templates influenced search in a particular way, by reducing the likelihood that MP re-selected previously inspected objects or locations. This may be because search based on action templates helped MP to generate a better memory of previously search objects or locations.

We conclude that action-defined templates exist, that they are activated by affordances from objects (in Experiment 1a but not 1b), and they can help a patient establish a memory representation for previously searched objects or locations (Experiment 2). We discuss the implications of the results for understanding the links between perception and action, and for understanding different forms of unilateral neglect.

[1]When 1 target must be detected via serial search, N/2 items will need to be searched on average (where N = the number of items present, and is held constant). When 2 targets must be detected, the number of items searched on average will be N/2 + (N/2)/2. When 3 targets must be detected the number of items searched on average will be: N/2 + (N/2)/2 + {(N/2)/2}/2, and so forth. With 10 item arrays we predict that the number of items searched to detect 1, 2, 3 or 4 targets will on average be: 5, 7.5, 8.75 and 9.4.

## 4. The case

MP (d.o.b. 19.1.47) was a formerly left-handed toolworker who suffered an aneurysm of the right middle cerebral artery in 1992, resulting in cerebral artery occlusion and infarct. There was a mild left hemiparesis for his upper limb. MRI and SPECT scans showed that there was damage to fronto-tempero-parietal regions of his right hemisphere, including the inferior frontal gyrus, the superior temporal gyrus, the supramarginal and angular gyri and the post central gyrus (see [10, 20]). Figure 1 presents slices from an MRI scan on a standardised brain atlas. MP presented with aspects of unilateral left neglect, particularly in tasks requiring him to scan across cluttered visual arrays. On the standardised Behavioural Inattention Test (BIT) [37] he scored 94/146 on the conventional subtests, showing particularly poor performance on line crossing, star cancellation and letter cancellation tasks, where there were omissions of stimuli on both the far left and right sides of the page. There was also evidence of neglect in line bisection, particularly when the task involved pointing to the centre of the line [10]. There were few errors in reading single words, but whole words were omitted from the left side of the page when reading text (12 errors across 5 pages). There were also problems in detecting the facial identity and gender of the left side of chimeric faces (scoring 0/20 at identifying the left-side face and 10/22 [chance] at judging its gender). There was also evidence of a mild perceptual impairment on the VOSP [36], where he was in the lowest 5% of the population on the dot counting, position discrimination and number location sub-tests. Isolated objects and pictures could be named relatively well (70/76 on naming the full set of line drawings from BORB [29]) and there were no major problems in dealing with overlapping perceptual features (15/18 items named correctly with both overlapping and non-overlapping items from BORB). On the Warrington Recognition Memory test there was a general lowering of performance, particularly for faces (35 vs. 25/50 for words and faces), consistent with right hemisphere pathology. Verbal short-term memory was impaired (digit span 4) and there were also problems in mental arithmetic. These last problems are unusual after right hemisphere damage but may be due to MP having crossed laterality.

### 4.1. Experiment 1: Varying the numbers of distractors

Experiment 1 contained two parts: (a) with objects and (b) with words, in the search arrays. For both types

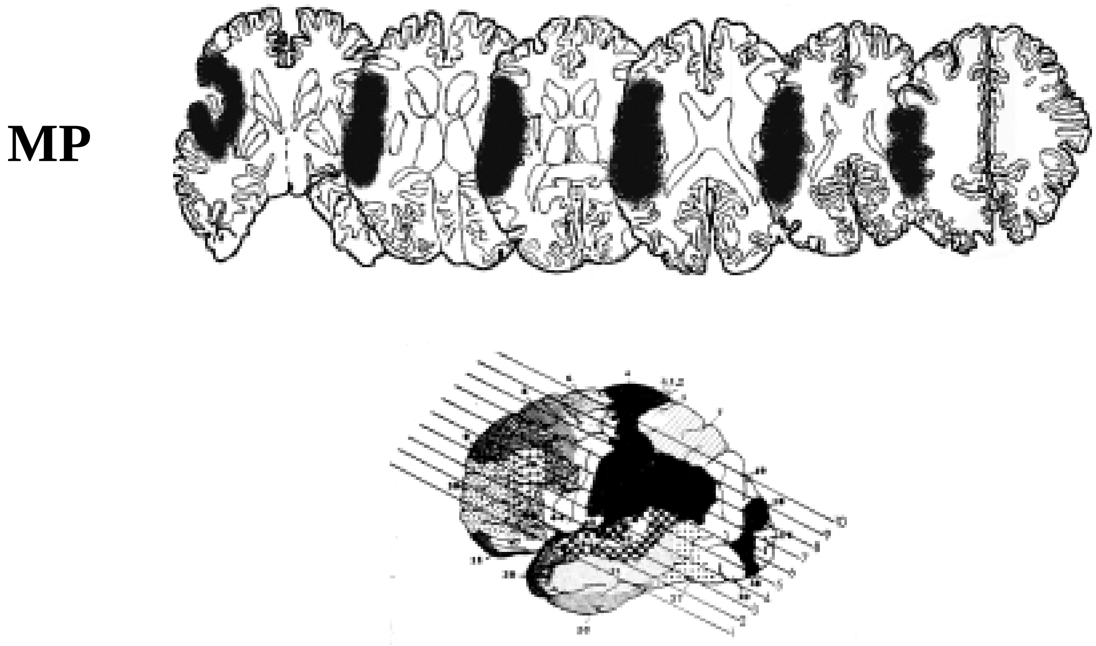

Fig. 1. Interpretation of MP's MRI scan drawn on standardised slices from Gado et al. [11]. The 10 slices used are illustrated at the bottom of the figure. Only slices 3–8 are depicted here. The left of each slice represents the right hemisphere.

of array, MP was cued to search for a target defined either by its name or by an action ('find the cup' vs. 'find an object you could drink from'). Humphreys and Riddoch [20] found that the advantage for action-defined templates occurred only with object arrays, and attributed this to affordances being activated by objects but not by words. We sought to replicate this result, but now in the context of varying numbers of distractors.

### *4.2. Experiment 1a: Object arrays*

#### *4.2.1. Method*

MP sat at the centre of a table 120 cm wide by 60 cm deep. His task was to point to a target object defined by the experimenter. Prior to each trial a cardboard shield was placed in front of an array of either 6 or 10 objects. The objects were arranged in pre-set locations, 5 to the right and 5 to the left of MP's midline. The items assigned to the positions were chosen at random for each trial, and the positions used for the 6-item trials were chosen randomly from those used for the 10-object arrays, with the proviso that 3 fell to the right and 3 to the left of MP's midline. On each side of midline, the flanking and middle positions were at a depth of 40 cm and the 2 inter-leaved positions were at depths of 50 cm. Between trials the objects were re-arranged and assigned randomly to new locations relative to the prior trial, with the constraint that the target had to fall no more than once in a given location across the block. For both array sizes, each object from the set of 10 was chosen as a target once per block. The conditions (2 array sizes $\times$ 2 cueing instructions) were presented in blocks of 10 trials, with the order of the blocks counterbalanced across test sessions (ABCD, BCDA, CDAB and DABC). Consequently, there were 40 trials per condition, with each object being cued as a target 4 times. The objects were: cup (red), hammer (wooden), hairbrush (blue), pen (black), key (brass), scissors (light silver), teapot (brown), screwdriver (yellow), hacksaw (dark metal), stapler (green). All objects were positioned so that the part to-be-grasped in an action (e.g., the handle of the cup) was oriented towards the ipsilesional (right) hand. Each trial began with the instruction from the experimenter. The action definitions stressed the functional action that would be performed with a target ('find the object you would drink with, hit a nail with, straighten your hair with' etc.). In the name definition condition MP was given the name of the target and asked to imagine what it looked like when trying to find it. In a pretest MP was able to name all the individual objects used in the study, when shown to him one at a time. Following the instruction, MP indicated that he was ready to start search and the divide between MP and the objects was lifted; search

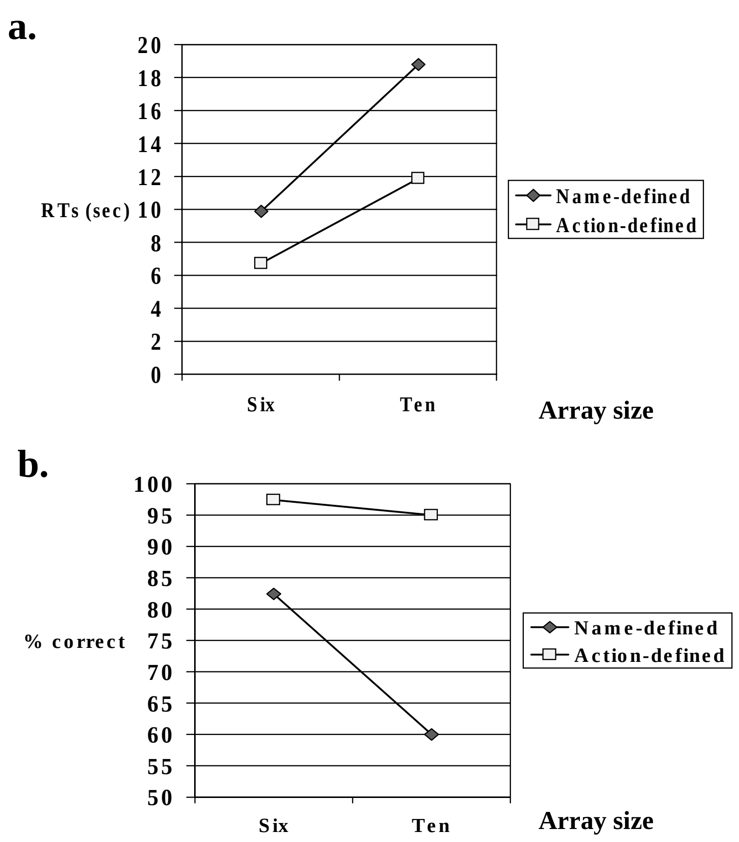

Fig. 2. (a) Mean correct RTs (sec) and (b) Mean percentage correct responses for MP in Experiment 1a (search array of objects).

was timed by stopwatch by one of the experimenters. Performance was also video-taped and timings were verified by an independent judge.

*4.2.2. Results and discussion*

The data were analyzed by summing (for accuracy) or averaging (for RT) the results for each object across the test blocks (so for accuracy there was a maximum score of 4 per object, and RTs were based on an average derived from a maximum of 4 correct responses for each object). Figure 2 presents the mean RTs and percentage correct responses, over targets.

Analyses of variance (ANOVAs) were performed with target object as a random factor; there were two repeated measures factors: cue (target defined by name or by action) and array size (6 or 10).[2] For RTs there were reliable main effects of cue and array size, $F(1,9) = 57.85$ and $69.72$, both $p < 0.001$. There was also a significant interaction, $F(1,9) = 8.38,\ p < 0.01$. RTs were slower for targets cued by name over those cued by action, they increased with the array size, and the increase with the array size was larger for targets cued by name than for those cued by action.

The accuracy data followed a similar pattern. There were reliable main effects of cue and array size, $F(1,9) = 25.71$ and $22.5$, both $p < 0.01$, and a reliable cue x array size interactions, $F(1,9) = 7.58,\ p < 0.02)$. More targets were detected when cued by action, and when the array size was smaller. The decrease in accuracy with the array size was most marked for targets cued by their name.

The errors reflected failures to detect the target on the trial, when MP reported that he could not find the target. Of the 26 trials where this occurred, 20 had targets on the left of the array and 6 had targets on the right.

The basic result replicated the findings of Humphreys and Riddoch [20], that there was an advantage when MP had to detect a target object defined by action relative to when it was defined by its name. MP was con-

[2]Unfortunately there were too few RTs per item to enable ‘side’ (left or right of midline) to be included as a further factor in the ANOVA.

siderably faster at finding action-defined targets, and he also made fewer 'misses', where he failed to detect the target. The data support the idea that action-defined templates can exist in addition to those that specify the perceptual properties of objects, and that MP is better able to use a template defined in terms of an action than a template specifying the perceptual properties of objects. The advantage for action-defined targets also increased when larger arrays of objects were presented. It is unlikely that these effects were determined by the generality of the instruction – contrasting definitions of a specific name relative to definitions of a more general action, that could be applied to a range of specific objects (e.g., all of which could be drunk from). In our previous study, we found that MP's performance was similar when cued by colour that could apply to a number of objects ('find the red object') and when cued by a specific name ('find the cup') [20]. It seems less important whether the cue was general or specific, and more important whether the cue defined an intended action rather than the perceptual properties of an object.

Experiment 1b tested whether the action-advantage in Experiment 1 was particular to when MP searched objects, as would be expected if action-defined templates are activated by affordances. In Experiment 1b, the objects in the search arrays were replaced with cards with the names of the objects printed on them.

### 4.3. *Experiment 1b: Word arrays*

#### 4.3.1. *Method*

The Method was exactly the same as for Experiment 1a, except that the stimuli were presented as words on cards rather than as real objects. Each card was 15 cm long by 3 cm high, and it was folded over like a place-card with one word written across it, facing MP. Words were printed in Times size 28. In a pre-test MP was able to read all the names aloud, when each card was presented individually at the further distances used in the study.

#### 4.3.2. *Results and discussion*

Figure 3 gives the mean correct RTs (sec) and percentage correct detection responses over items.

For RTs there was a reliable main effect of array size, $F(1,9) = 71.33,\ p < 0.001$. Neither the main effect of cue nor the cue x array size interaction was significant (both $F < 1.0$). The same findings occurred with the accuracy measure. Array size was significant ($F(1,9) = 19.29,\ p < 0.001$), whilst the effect of the cue and the cue x array size interaction did not approach significance (both $F < 1.0$).

The advantage for search cued by action rather than by the name of the target, in Experiment 1a, was eliminated here. MP was equally poor at detecting targets defined by action and by their name, when the targets were words. He made 49 detection errors, 30 with targets on the left of the arrays and 19 with targets on the right. He tended to neglect the left side of arrays, whether cued by the target's name or its action. We conclude that cueing for action is effective only with objects, whose properties are functionally related to their use and which can activated action-defined templates by means of affordances. We also note that this result helps to rule out one alternative account of the action-advantage in Experiment 1a, which is that the advantage was due to the action cue being more attentionally arousing than the name cue. Neglect can be reduced when arousal levels are increased (see [33]). However, if arousal from the instruction were important, the same results ought to occur with word and object targets. They did not.

### 4.4. *Experiment 2: Varying the number of targets*

Experiment 1a demonstrated an interaction between cueing a target by action and the size of the search array; the advantage for action- over name-based templates increased at the large array size. As we pointed out in the Introduction, an additive relationship between cue and array size could be given an unambiguous interpretation; unfortunately, an interaction, such as we found, cannot. An interaction between cue type and array size could occur because the cues influence the search process directly, or because the cues affect the time taken to match each object against the memory template in a serial search process. To provide a stronger test of why the instructions had such an effect on MP's search in Experiment 2, we varied the number of identical targets present and required him to search for a specific number of targets. For a serial search process, the time taken to detect the required number of targets should increase as the number of targets increases, but this effect of target number should be asymptotic (i.e., the RT increments with each target should decrease as more targets are added). If the cues influence only the time to match to the memory template, in serial search, then an asymptotic pattern of search should be found across all cue conditions (and the effect of cue may even combine additively with that of target number).

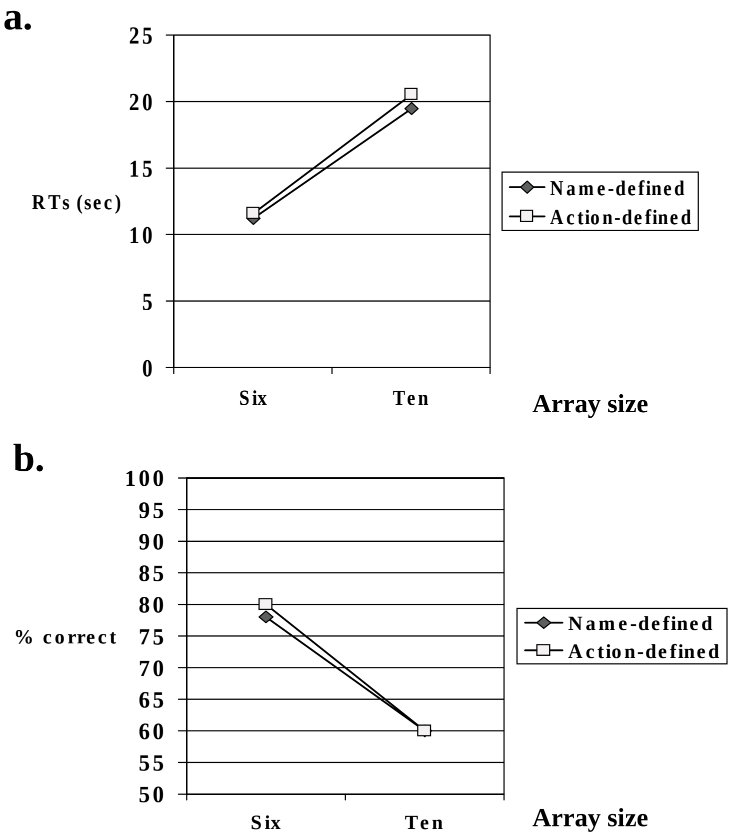

Fig. 3. (a) Mean correct RTs (sec) and (b) Mean percentage correct responses for MP in Experiment 1b (search array of words).

*4.4.1. Method*

The procedure was generally the same as for the larger array sizes in Experiment 1a, except that MP had to point to the presence of varying numbers of targets across different trial blocks. Each array always contained 10 objects, and the task required the detection of 1, 2, 3 or 4 targets. The targets, when present, were identical replicas (same colour and same size) and they replaced up to 4 distractor objects. The distractors to-be-replaced were chosen semi-randomly with the following provisos. When 1 target was present, it appeared equally often on either side of MP's midline; when 2 targets were present 1 fell on each side of his midline; when 3 targets were present 2 appeared on the left and 1 on the right on half the trials (and vice versa on the other half); when 4 targets were present, 2 appeared either side of the midline. There were 10 trials per target number block, with each possible object acting as the target once per block. The order of the blocks was counter-balanced across 4 test sessions, so that overall each object appeared as a target 40 times in each target-number condition. In each session there was 1 block of trials with the target defined by its name and 1 block with the target defined by its action. The order of the cue conditions was reversed across test sessions. MP was told the number of targets present at the start of each block and a trial was only scored as correct if MP pointed to all the individual targets in the array.

*4.4.2. Results*

Figure 4 presents the mean correct RTs (sec) and the percentage of correct target detections as a function of the number of targets present and the cue.

The RT data were analyzed in a two-factor repeated measures ANOVA, with cue and target number as fixed effects and items treated as a random factor. Missing data were treated by substituting the mean RT over items in that condition. The main effects of target number and cue were reliable, $F(3,27) = 64.96$ and $F(1,9) = 79.67$, both $p < 0.001$. There was also a significant target number x cue interaction, $F(3,27) = 14.27$, $p < 0.01$. RTs increased when name- relative to action-definitions were used to cue

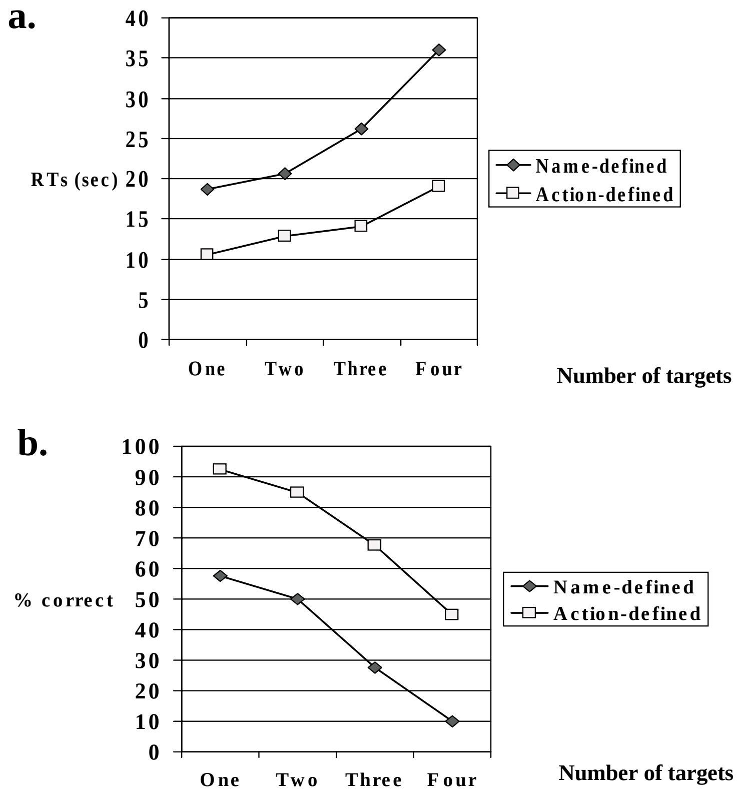

Fig. 4. (a) Mean correct RTs (sec) and (b) Mean percentage correct responses for MP in Experiment 2 (search arrays of objects, with varying numbers of targets).

targets, and they increased when more targets were present. The RT functions were exponential rather than asymptotic, however, since the size of the RT increment increased as more targets were added. The interaction arose because there were larger RT increases with more targets in the name-cue condition compared with the action-cue condition.

In an equivalent analysis of errors there were reliable effects of the number of targets and the cue, $F(3,27) = 67.03$ and $F(1,9) = 13.03$, both $p < 0.01$. The interaction was not reliable ($F < 1.0$). Accuracy was higher for action-defined relative to name-defined targets, and accuracy decreased as more targets were added. Any interaction between these factors may have been obscured by a floor effect in the 4 target condition.

Overall MP made 174 errors (from 320 trials). On 1 item trials he made 20 errors, all of which arose because he was unable to find the target on a given trial. 16 misses were for left-side and 4 for right-side targets. When more than 1 target was present he again made errors by missing targets, though errors also occurred when he pointed to the same target on two or more occasions. There were 26 errors with 2 targets, 22 misses (17 left, 5 right) and 4 where he re-counted one of the targets. With 3 targets there were 28 misses (20 left and 5 right), and 14 errors due to re-counting. With 4 targets there were 58 errors, 36 misses (24 left and 12 right) and 22 re-counts. On trials with 2 or more targets there were 55 misses and 30 re-count errors in the name-definition condition; there were 31 misses and 10 re-count errors in the action-definition condition. Overall there were more misses for targets on the left of midline than would be expected by chance (Chi square $(1) = 12.42$, $p < 0.001$). MP never made an error by pointing to a distractor.

*4.4.3. Discussion*

The results again demonstrate that MP was better able to detect targets defined in terms of their action than targets defined by their name, and he tended to miss targets that appeared on his left side. The benefit for the action- over the name-definition condition supports the argument for memory templates based on actions.

How do these action-templates facilitate MP's performance? MP performed very slowly, often taking up to 20 sec to detect a single target and 36 sec to detect four. With such long RTs, it is difficult to argue for a parallel search process and it is likely that he searched displays serially. However, the data indicate that any serial search was highly inefficient. As we have pointed out, a serial search account predicts that there should be asymptotic increases in RTs as more targets need to be detected for the task. This account assumes, though, that there is a memory for objects/locations already searched, so that items are not re-inspected. If this is not the case, then RTs need not increase asymptotically. Indeed, if the probability of re-searching at least one target increases with the number of targets present, then RTs can increase exponentially, as we found. Re-inspections would occur if MP has difficulty in establishing a memory of where he has searched. This suggestion is reinforced by the kinds of error MP made when more than 1 target was present. In addition to missing some targets, MP made errors by re-counting the same target (pointing to the same target on more than one occasion whilst also failing to point to at least one of the targets present). Previous studies have demonstrated that patients with fronto-parietal damage can show poorly organised search, with frequent re-sampling of locations already inspected [4,7]. This impairment may relate in part to a deficit in creating token representations for each object, linked to the locations they occupy (cf. [2]). The same appears to hold for MP. The novel result here is that the likelihood of re-sampling errors decreased when MP was cued by an action-definition relative to when he was cued by the target's name; consequently the exponential increases in RTs with target number were less pronounced for action-compared with name-defined targets. Thus one possible reason for the benefit for action cues was because MP was better able to maintain a record of his search when stimuli activated an action-based template. An alternative is that partial activation of action-templates from the multiple targets in the displays could have decreased the likelihood that the same target was re-sampled, since activation of the template from a new item may have helped MP sample the display more fully. When this activation was decreased (with a name template), there was increased re-sampling. In either case, search improved.

## 5. General discussion

We have reported two experiments that have confirmed that, in a patient showing symptoms of unilateral neglect, there can be better detection of targets defined by their usual action than targets defined by their name (see also [20]). This advantage for action over name definitions is confined to when search arrays contain objects and it is eliminated when arrays contain words (Experiment 1b). The data are consistent with the idea that action-defined templates can be used in search, and with these templates being activated by affordances from objects.

In Experiment 1a, the advantage for action over name cues increased when arrays contained more distractors. In Experiment 2 this same advantage increased as more targets had to be detected. In addition, MP's RTs increased exponentially in all conditions as more targets had to be counted (though the total array size remained constant). This last finding suggests that MP was impaired at establishing a memory record for objects/locations already searched, so that there was an increased probability of re-sampling the same objects on trial blocks on which more targets were present. Indeed, on many trials with 2 or more targets present MP's errors were due to pointing to the same target on more than occasion. This suggests that MP may have been helped by using an action-defined template because these templates were used to form a more stable map of locations already sampled. Most theories of search assume that re-sampling is minimized by inhibition of recently inspected locations and/or objects (e.g., see [8,27]), and this may in part depend on processes intimately linked to preparation of a motor response to an object at a given location (cf. [28]). Use of an action-defined template may generate stronger motor-based inhibition of old objects or locations, as the recipients of intended actions. With stronger inhibition of old objects or locations, more new stimuli can be detected. Alternatively, partial activation of these templates from the other objects present could have helped MP re-direct his search away objects or locations already sampled. The net effect of the action-defined template is to facilitate search.

MP manifested a form of visual neglect in which he missed targets predominantly on the side contralateral to his lesion. For him, neglect could be improved by basing search upon action-templates, which we propose are activated from affordances. What defines such affordances? We suggest that affordances are defined in terms of a correspondence between the visual properties of objects and an intended action. Both the visual properties of the object have to be appropriate, and the intention for action present, in order for an affordance to arise. Hence, for MP, the mere presence of a target object was often insufficient for it to be detected when the target was defined by its name – the action template also had to be established. Similarly, when the visual properties of the objects were less appropriate for action, action-cueing was no longer effective. In our prior study the action-advantage with MP was eliminated when objects were re-oriented so that their handles faced away from him [20]. However, rotating the objects had no effect on search when targets were defined by their names. This last finding indicates that the effect of rotation was unlikely to be due to poor object recognition/access to semantics – the process we assume to operate when objects are matched against perceptual or name templates. Rather than this, the rotations appeared to reduce the direct correspondence between the handles of the objects and the action cued by the definition of the target; we refer to an affordance as this direct correspondence between the parts of objects and an intended action. Our prior study also found that the advantage for action-based templates extended to non-objects as well as objects, indicating that affordance are not necessarily based on learned associations with specific objects.

In other neuropsychological studies, we [31,32] have shown similar effects of affordances based on the orientation of a handle relative to the actor. Riddoch et al. [32] reported data from a patient, ES, with corticobasal degeneration and poor intentional control of action. The task was to grasp a cup with the hand appropriate to the location where the target was presented (e.g., using the left hand if the cup appeared to the left of her body). Despite being able to repeat back the task instructions, and despite showing good discrimination of the location of the target, ES made errors by grasping the cup with the hand compatible with the cup's handle rather than the task instructions (e.g., using her right hand to grasp a left-side cup with a handle on the right). Interestingly, these errors were reduced by changing the goal of the task, to pointing rather than grasping the handle of the cup. Apparently the object feature that elicited the errors (the cup's handle) had to be compatible with the task goal (grasp rather than point to the target) in order to generate the incorrect response. If the cup was inverted the errors were also reduced, though grasp responses were then sometimes made to the bottom of the cup. Inverted cups could still be recognized semantically, but they are more 'graspable' at their bottom rather than at their handle. Thus MP was unable to use novel task requirements to overrule actions that were congruent with both the visual features of the target (the orientation of the cup with respect to her hand) and the task goal (grasp rather than point). Incorrect responses were based on the affordance of the objects for grasping.

MP appears to be impaired at using perceptual templates to direct search, given his problems in finding targets cued by their name. Perceptual templates may normally influence search by biasing ventral recognition processes to favour targets over distractor objects (cf. [6]), though the templates themselves may be established in areas of frontal cortex (see [24]). MP has an extensive brain lesion, that includes regions of superior temporal cortex as well as the right frontal and parietal lobes. It may be that the damage to his right, superior temporal cortex prevents MP from using a perceptual template to direct search. Consistent with this, in other patients with neglect but without damage to the superior temporal cortex, perceptual templates can still be used to direct search (see [20]). In contrast to the evidence for perceptual templates being implemented within the ventral visual stream used for object recognition, there is some suggestion that affordances activate actions through a separate route. Thus patients with temporal lobe damage, who show semantic dementia and poor performance on standard tests of object recognition, can nevertheless make accurate decisions about how objects can be used [15]. Similarly patients with 'optic aphasia' can be impaired at making semantic judgements about visually presented objects whilst demonstrating relatively preserved gestures [17, 29]. Hodges et al. propose that actions can be activated through a 'pragmatic' dorsal route, separate from more ventral visual areas mediating the retrieval of semantic knowledge about objects. For MP, this pragmatic visual route may continue to operate despite his lesion, so that he can respond to affordances activated by objects in search tasks.

There are other cases of neglect who appear to show the opposite pattern of deficit to MP. For example, patients with 'motor neglect' may make appropriate perceptual judgements to objects whilst being impaired at moving into the impaired spatial region (e.g. [14,23]). MP, in contrast, showed impaired detection (particularly for contralesional stimuli) when cued perceptually (by the object's name) relative to when cued in terms of the action that might be performed. In other studies we have demonstrated that MP made more neglect errors in bisection when pointing rather than grasping the centre of a rod. However, the resolution of neglect in the grasping task occurred only when grasping was performed with on-line visual feedback and even then it was present only in the end part of the reach trajectory [10]. To account for these bisection results, we argued that the initial stages of the grasping action were based on an impaired perceptual representation of space. On-line visual feedback was used to help correct this distorted representation when a grasp response was made, but not when a pointing response occurred (perhaps because visual feedback is more important for grasping). In the present study, MP's poor performance when cued with the object's name may again reflect an impaired perceptual representation, which can be by-passed when he uses an action-defined template to respond to affordances. In patients showing motor neglect, there may be a relatively intact perceptual representation along with an impaired ability to respond in motor action to affordances from the environment.

## Acknowledgements

This work was supported by grants from the Medical Research Council and the Wellcome Trust to both authors. We thank MP for his kind participation in the study and Dr V. Chavda for help in interpreting the scans.
